# Prolonged Proofing Modulates the Acrylamide Content, Nutritional and Functional Characteristics of Pumpkin (*Cucurbita maxima* Plomo) and Soft Wheat Composite Bread

**DOI:** 10.3390/foods14030437

**Published:** 2025-01-29

**Authors:** Durim Alija, Remigiusz Olędzki, Daniela Nikolovska Nedelkoska, Agata Wojciechowicz-Budzisz, Ewa Pejcz, Vezirka Jankuloska, Gafur Xhabiri, Joanna Harasym

**Affiliations:** 1 Faculty of Technology and Technical Sciences Veles, University St. Kliment Ohridski-Bitola, Dimitar Vlahov 57, nn 1400 Veles, North Macedonia; durim.alija@uklo.edu.mk (D.A.); daniela.nedelkoska@uklo.edu.mk (D.N.N.); vezirka.jankuloska@uklo.edu.mk (V.J.); 2 Faculty of Food Technology and Nutrition, University of Tetova, Str. Ilinden, nn 1200 Tetova, North Macedonia; gafur.xhabiri@unite.edu.mk; 3 Adaptive Food Systems Accelerator-Science Centre, Wroclaw University of Economics and Business, 53-345 Wroclaw, Poland; remigiusz.oledzki@ue.wroc.pl; 4 Department of Biotechnology and Food Analysis, Wroclaw University of Economics and Business, Komandorska 118/120, 53-345 Wroclaw, Poland; agata.wojciechowicz-budzisz@ue.wroc.pl (A.W.-B.); ewa.pejcz@ue.wroc.pl (E.P.)

**Keywords:** acrylamide, bread, *Cucurbita maxima* Plomo, proofing time, antioxidant activity, polyphenolic compounds, texture profile

## Abstract

Acrylamide formation in bread products poses health concerns, necessitating strategies to reduce its presence while maintaining nutritional value. This study investigated how different concentrations of pumpkin flour (*Cucurbita maxima* Plomo) and prolonged proofing times affect acrylamide content and bread characteristics. Composite bread samples were prepared with varying pumpkin flour shares (0–20%) to soft wheat flour using two proofing times (60 and 120 min). The study analyzed quality features, crust and crumb color, antioxidant activity, total polyphenolic content, reducing sugars, and acrylamide content of the resulting breads. Extended proofing (120 min) reduced acrylamide levels in the crust from 220 to 150 units in 20% pumpkin flour bread compared to 60 min proofing. Control bread showed the highest specific volume (2.40 ± 0.01 cm^3^/g) after 2 h of proofing, while 20% pumpkin flour addition decreased it to 1.69 ± 0.02 cm^3^/g. Initial hardness increased from 6.8 ± 1.5 N in the control to 14.3 ± 1.5 N in 20% pumpkin flour bread after 1-h of proofing. Water activity decreased from 0.966 ± 0.002 in the control to 0.945 ± 0.004 in 20% pumpkin flour samples with 2 h proofing. Optimal results were achieved with 5–10% pumpkin flour substitution combined with two-hour proofing, balancing improved nutritional properties and reduced acrylamide formation while maintaining acceptable bread quality parameters.

## 1. Introduction

Acrylamide, a substance that is created through high-temperature procedures used in food processing, has been labelled by the International Agency for Research on Cancer (IARC) as a probable human carcinogen since 1994 [1]. Although the data regarding the acrylamide toxicokinetics in humans are scarce, various toxicological investigations suggested that after its absorption through dermal, respiratory, and digestive systems, the acrylamide is widely distributed to different tissues and may cause gene mutation, negative effects on the reproductive system, and damage to the nervous system [2]. Acrylamide forms during the complex Maillard reaction, a chemical process involving reducing sugars and amino acids that predominantly determines the color, flavor, and texture of baked products [3]. Several research findings confirmed asparagine’s role as a major precursor to the development of acrylamide in heat-treated foods based on cereal [4,5,6]. Over the past decades, health risks associated with acrylamide have been documented [7], emphasizing the need for strategies to mitigate its presence in food. It has genotoxic potential through glycidamide, a chemically reactive epoxide of acrylamide in animals.

Breads are the primary product of the cereal-based food category, which is the largest food group consumed. Because of its high consumption rate, bread is one of the main contributors to daily acrylamide intake in many countries. The bread’s high carbohydrate and protein content and the baking process make it susceptible to acrylamide formation. Several routes have been suggested for acrylamide formation in bread [8]. However, the quantity and ratio of precursors involved in acrylamide formation, the flour quality, application of enzymes, the processing methods (thermal and innovative), and processing conditions (type of fermentation, temperature, heating time, pH, water content and activity, and additives) may affect the formation of acrylamide [9]. The acrylamide formation is exceptionally typical for the bread crust, where the temperature reaches higher values during baking than in the bread crumb [10,11,12]. Hence, developing new concepts for acrylamide reduction in breads is necessary for food sectors and safeguarding human health. In general, the strategies to minimize the acrylamide formation in cereal products are focused on three main approaches: (a) elimination or replacement of precursor substrates, (b) modification of treatment conditions, and (c) elimination or reduction in acrylamide after formation [13].

Several studies were concerned with monitoring and exploring factors influencing acrylamide formation in bakery products to establish procedures for acrylamide reduction [14]. There is evidence for the role of antioxidants in reducing acrylamide content in bread [13]. Additionally, dietary fiber has been shown to reduce moisture loss during baking [15], reducing acrylamide development [16,17]. Prolonged yeast fermentation, in which yeasts consume asparagine, is another potential method of lowering the quantity of acrylamide [18,19]. The current state of the art is summarized in the document published by Food Drink Europe in 2019 [20].

Despite the importance of decreasing acrylamide, it is necessary to consider the nutritional and functional characteristics of the bread since the product must meet all consumer’s requirements. Today, the introduction of vegetables, as essential sources of diverse bioactive metabolites, to the wheat matrices is a frequently chosen approach in food reformulation. Hence, adding raw vegetable materials to the recipe aims to improve the product’s nutritional quality and functional properties. In contrast, the potential reduction in acrylamide content in the product is a desired additional benefit. Pumpkin (a member of the genus *Cucurbita*) is a widely cultivated vegetable known for its nutritional value and health-promoting effects. Numerous studies have demonstrated that the antioxidants in pumpkin, by inhibiting free radicals, lower the risk of cancer, heart disease, and neurological disorders [21,22,23]. Hence, it has been shown that pumpkin powder, rich in components such as carotenoids, water-soluble vitamins, phenolic compounds, dietary fibers, and minerals [24,25], can replace white flour in bread [26,27], which provides substantial beneficial nutrients but also changes the texture and sensory properties of the fortified product [28]. The nutritional and techno-functional characteristics and its bioactive profile indicate that the pumpkin could be utilized in developing sustainable and innovative functional food products [29,30]. There are some studies investigating the influence of the addition of pumpkin flesh or seeds on bread properties. Hoxha et al. (2023) [31] have proven that adding pumpkin flour to the production of breads decreases the volume and specific volume of loaves. Aukkanit & Sirichokworraki (2016) [32] showed that adding pumpkin flour up to 30% for bread production affects the texture profile, increasing the hardness and chewiness and reducing the cohesiveness of bread. Adding pumpkin powder to bread increases the redness (a) and yellowness (b) of the bread [33], its total phenolic content [34], and β-carotene content in some baking products [35].

According to Eun-Sun & Tae (2022) [36], adding pumpkin powder may help prevent the formation of acrylamide by changing the chemical composition of the dough and decreasing the amount of asparagine, a crucial precursor in the formation of acrylamide. The fermentation period can affect the amount of acrylamide that forms in bread. Due to the breakdown of precursors during fermentation, prolonged proofing has been associated with reduced acrylamide in bread [18] and improved rheological properties. Zhou et al. (2022) [37] suggested that sourdough fermentation with appropriate strains can be used as an advantageous technology to reduce the acrylamide content of bread, while Khorshidian et al. (2020) [38] indicated that probiotics can decrease acrylamide formation by producing asparaginase and removing produced acrylamide via cell wall peptidoglycan.

Considering that the food industry needs to introduce strategies to mitigate the formation of acrylamide without compromising the quality of the product, this study aimed to validate the capacity of prolonged proofing time of soft wheat pumpkin flour composite bread to reduce the acrylamide content of soft wheat pumpkin flour composite bread while maintaining acceptable quality features and nutritional composition. For this purpose, wheat-pumpkin bread samples (0–20% pumpkin flour) were prepared using two proofing times (60 and 120 min) before baking. Quality features of bread, like bake loss, specific volume, external appearance, water activity, porosity, and texture, were evaluated, as well as crust and crumb color, antioxidant activity, total polyphenolic content, and reducing sugars and acrylamide content.

## 2. Materials and Methods

### 2.1. Raw Materials and Ingredients

The soft wheat flour bread type (T 750, Stoisław, Poland; fat 1.8%, of which 0.4% was saturated fat, carbohydrates 68%, protein 12%, fiber 2.9%, and ash 0.64%, moisture content-13.8%, and the pumpkin flour, *Cucurbita maxima* Plomo, Kamenjane, North Macedonia, 8.04 g protein, total dietary fiber—39.2 g, insoluble dietary fibre—24.8, and soluble dietary fibre—14.4). The pumpkin was sliced into small pieces and then shredded entirely using a grater after removing the stem, peel, and seeds. Once grated, pumpkin flesh was dried in an oven at 45 ± 2 °C for 24 h until reaching less than 10% moisture content and milled (35,000 rpm, HC-350, Chemland, Stargard, Poland). The flour blends were prepared using a rotating drum mixer (TM100, Vevor, Guangzhou, China). Additional recipe components, such as baking yeast and salt, were purchased in a local supermarket in Wroclaw (Poland).

### 2.2. Breadmaking

Breads were prepared using a recipe where the flour blends were considered 100% of the flour content, with 2% salt, 2% yeast, and 55% water (calculated per flour weight) for all the blends. A total of five types of bread, differing in flour composition, were prepared as follows: 100% soft wheat flour (control), 95:5 (*w*/*w*) blends of soft wheat flour and pumpkin flour, 90:10 (*w*/*w*) blends of soft wheat flour and pumpkin flour, 85:15 (*w*/*w*) blends of soft wheat flour and pumpkin flour, and 80:20 (*w*/*w*) blends of soft wheat flour and pumpkin flour. Based on prior expertise and initial testing, these mixes were developed. The dough was kneaded for 5 min in a 1000 W dough mixer at speed level 5 (MUM58231, BOSCH, Stuttgart, Germany). Approximately 100 g of dough was placed into bread trays for shaping. After the dough was divided into trays, they were fermented at 35 °C for 1 h and 2 h at 82% humidity. The breads were then baked in a steam-convection oven (HENDI 227077, Rhenen, The Netherlands) at 180 °C for 30 min and cooled for 1 h. Subsequently, the quality characteristics, antioxidant activity, total polyphenolic compounds, and acrylamide content were measured below in all samples (with 1 and 2-h proofing). Samples of the bread loaves were stored in a refrigerator (4 °C), wrapped in parchment paper, for 7 days, and some analyses were repeated. Five types of bread were obtained from the formulations, with four trays per formulation (four replicates for each).

### 2.3. Quality Features of Breads

#### Weight Loss and a Specific Volume

Baking loss was measured by weighing the samples before baking (W_s_) and 1 h after baking (W_b_), and weight loss (W_w_) was measured after 7 days. The following formula (Equation (1)) was used to obtain the baking/weight loss percentage:

Bake loss/Weight loss [%] = [(W_s_ – W_b/w_)/W_s_] × 100 (1)

One hour after baking, the volume of bread loaves (V) was measured using a 3D scanner (Matter and Form v2, Toronto, ON, Canada) using the Quick scan program to capture 3D scans with the volume and surface estimated. Each sample was measured three times. The following formula (Equation (2)) was used to obtain the specific volume:

Specific volume [%] = V/W_b/w_ [cm^3^/g] (2)

### 2.4. Physicochemical Characterization of Breads

An AquaLab 3TE analyzer (Decagon Devices, Inc., Pullman, WA, USA) was used to measure water activity (aw). The color parameters are measured using the CIE*Lab* scale (L*, a*, b*) with a reflective spectrophotometer (TS7036, #nh, Guangdong, China) on the crust and cross-section crumb (40–45 mm width). Chroma, hue angle, browning index, and whitening index were calculated for the bread’s crust and crumb. Analysis was carried out in quadruplicate. The results were reported as mean ± standard deviation. A D65 standard illuminator and a − 2° standard observer were used.

For crumb and crust, the following formulas were used to determine the browning index (BI) (Equation (3)) and the whiteness index (WI) (Equation (4)):

WI = 100 − √(100 − L*)^2^ + (a*^2^) + (b*^2^) (3)

BI = (100 × (x − 0.31))/0.172(4)

where,

x = (a* + 1.75L*)/(5.645L* + a* − 3.012b*) (5)

#### 2.4.1. Texture Profile Analysis of Bread

The texture of the bread was assessed 1 h after baking and after 7 days of storage using a double compression test (texture profile analysis) with a texture analyzer FC20STAV500/500 (AXIS, Gdansk, Poland). Data were acquired by AXIS FM v.2_18 software, as was previously documented in Olędzki et al. (2023) [39]. While cohesiveness, springiness, and chewiness were determined from the peaks, hardness (N) was the force at maximal deformation. Analysis was carried out in quadruplicate. The mean ± standard deviation was used to report the results.

#### 2.4.2. Measurement of Bread Porosity

A bread porosity study was performed using the methods outlined [40] with a few adjustments. The bread loaf was cut in the middle, and the picture was acquired with a flatbed scanner (X1250, Lexmark, Lexington, KY, USA) 1 h after baking and after 7 days of storage. Image J software was used to analyze the resulting image. First, an 8-bit greyscale image of the bread was created from the original. Second, the image of the bread’s center, which measured 2 cm by 2 cm, was captured, and the software then processed the intercepted image to produce the bread’s binary image. Finally, the bread’s porosity value could be determined. Four replicate tests were carried out at every level, and the results were averaged.

### 2.5. Antioxidant Activity, Total Phenolic and Reducing Sugar Contents

#### 2.5.1. Extract Preparation

The crust and crumb of the baked bread were dried below 50 °C and then ground in a small laboratory grinder (Rommelsbacher EKM 100, ROMMELSBACHER ElektroHausgeräte GmbH, Dinkelsbühl, Germany). One gram of each bread from the crust and crumb (control, 5%, 10%, 15%, and 20%) was extracted with 5 mL of ethanol (99.99%) for the antioxidant activity (DPPH, ABTS, and FRAP) and total phenolic contents or with distilled water for reducing sugar contents. Samples were agitated for 1 h at 60 rpm on a radial stirrer (MX-RD PRO, ChemLand, Stargard, Poland), and then were centrifuged (15 min, 5000 rpm; MPW-350, MPW, Warsaw, Poland). After centrifugation, the supernatant was examined for total antioxidant potential (DPPH, ABTS), total reductive potential (FRAP), total polyphenol content (TPC), and reducing sugar content (Bernfeld method).

#### 2.5.2. Determination of Total Phenolic Compounds

The Folin–Ciocalteu reagent was used to quantify the total concentration of poly-phenolic compounds in samples. Folin–Ciocalteu reagent, when combined with anionic forms of phenolic compounds, creates a blue complex with maximum absorbance at 765 nm [41]. For each sample, a duplicate analysis was performed. A standard curve was created for gallic acid, and the amount of poly-phenolic chemicals was expressed in milligrams of gallic acid equivalents per gram of dry basis (DM).

#### 2.5.3. Determination of Antioxidant and Oxidoreductive Activities

The antioxidant capacity of samples was determined using the DPPH method, where the tested sample was mixed with a DPPH working solution and incubated for 20 min in the darkness and measured at 517 nm [41]. For each sample, a duplicate analysis was performed. The calibration curve was used to determine the total antioxidant activity of the tested material, which was then expressed in milligrams of Trolox equivalent (TE) per gram of dry matter (DM).

Sridhar et al. (2019) method was used to assess a bread’s crust and crumb anti-radical capability against cationic radical 2,2-azo-bis 3-ethylbenzothiazoline-6-sulfonic acid (ABTS•+) [42]. The stock solution of ABTS•+ was diluted with phosphate buffer (pH = 7.4, 0.2 M), and the blend’s extract was added to the working solution of ABTS•+ (incubation period—10 s) before the absorbance reading at 734 nm. For each sample, a duplicate analysis was performed. The total antioxidant activity was determined using a calibration curve, expressed in Trolox equivalents per gram of dry matter (DM).

The reducing power (capacity to reduce ferric ions Fe^3+^) of the bread’s crust and crumb samples was evaluated using the FRAP method by Re et al. (1999), with a few minor modifications [43]. The extract from the bread was combined with 1 mL of FRAP working solution. After stirring the mixture and letting it stand at 36 °C for 20 min, the absorbance at 593 nm was measured using a spectrophotometer (SEMCO, S91 E, Warsaw, Poland). For each sample, a duplicate analysis was performed. The total reducing activity was calculated using the calibration curve and then adjusted to micrograms of iron (II) sulfate equivalent FeSO_4_−7H_2_O per gram of dry matter (DM).

#### 2.5.4. Determination of Reducing Sugar Content

The Bernfeld method was used to quantify the reducing sugar concentration [44,45] with certain adjustments. A total of 0.25 mL of DNS reagent (1% of 3,5-dinitrosalicylic acid solution in 0.4 M NaOH) was combined with 0.5 mL of the tested extract. After five minutes of incubation in a boiling water bath, the mixture was cooled to about 50 °C. Then, 3 mL of distilled water was added to the reaction mixture, and the absorbance at 530 nm was measured. For each sample, a duplicate analysis was performed. The findings were expressed in glucose equivalent (GE) micrograms per gram of dry matter (DM).

### 2.6. Acrylamide Analysis

Acrylamide content was determined using ultra-high-performance liquid chromatography coupled with triple quadrupole mass spectrometry (UHPLC-MS/MS), according to Zhao (2019) [46]. One gram of sample was extracted with 10 mL of water and 10 mL of acetonitrile. The mixture was shaken for 10 min, followed by adding the QuEChERS salt mixture (4:1 MgSO4:NaCl). After centrifugation at 4000× *g* rpm for 6 min, 1 mL of the upper organic layer was diluted with 1 mL of water (dilution factor 20), or 2 mL of the upper layer was evaporated and reconstituted in 1 mL of water (dilution factor 5). The final extract was filtered through a 0.2 μm syringe filter before analysis. The UHPLC-MS/MS analysis was performed using an Agilent 1290 Infinity II LC (Agilent, Santa Clara, CA, USA) system coupled to a 6470A triple quadrupole mass spectrometer. Separation was achieved on a porous graphitic carbon column (100 × 3 mm, 5 μm) maintained at 60 °C. The mobile phase consisted of 0.1% acetic acid in water (A) and 0.1% acetic acid in methanol (B) at a flow rate of 0.25 mL/min. The injection volume was 5 μL. Mass spectrometric detection was performed in positive electrospray ionization mode with multiple reaction monitoring (MRM) of transitions *m*/*z* 72.1 → 55, 44, and 27 for acrylamide and *m*/*z* 75.1 → 58 for the isotopically labelled internal standard. Acrylamide was quantified using isotope-labeled internal calibration with 1/x weighting. The limit of quantification (LOQ) was 2.5 ng/g with a dilution factor of 5 and 10 ng/g with dilution factor 20.

### 2.7. Statistical Analysis

The results’ variance (ANOVA) and PCA analysis were evaluated with Statgraphics Centurion software (Centurion XVII.I version, StatPoint Technologies, Inc., Warrenton, VA, USA). ANOVA was performed with a previous normality of checked data using a *p*-value < 0.05 significance level. A three-way ANOVA for factors like day, proofing time, and sample was performed at a *p*-value < 0.05 significance level. PCA biplots and bootstrap graphs were prepared using the SRPlot platform [47].

## 3. Results and Discussion

During proofing, yeast fermentation produces CO_2_ bubbles that become trapped within the gluten network. At the same time, enzymatic activity modifies protein structures and starch properties, making the analysis of volume and surface critical for understanding dough development. Changes in the volume and surface of bread samples after different proofing times are presented in Table 1.

After one hour of proofing, the control bread exhibited a relatively high initial volume (189.9 ± 0.7 cm^3^), which increased significantly to 195.5 ± 1 cm^3^ after 7 days. Among the bread samples enriched with pumpkin flour, the 10% enriched bread had the highest initial volume (182.1 ± 1.4 cm^3^) among all samples in the one-hour proofing group. However, a clear declining trend in volume was observed as pumpkin flour concentration increased beyond 10%, with the 20% enriched sample showing the lowest volume (135.5 ± 0.1 cm^3^). In the two-hour proofing group, the control bread maintained a high volume throughout the storage period, starting at 178.5 ± 1.4 cm^3^ and reaching 195.1 ± 1.4 cm^3^ after 7 days. All pumpkin-enriched samples showed lower volumes than the control, with a consistent decrease in volume as pumpkin flour concentration increased. Like the one-hour proofing results, the 20% enriched sample exhibited the lowest volume values (137.3 ± 3.2 cm^3^ at day 0). Regarding surface measurements, both proofing times showed similar trends. The control and 10% enriched samples initially demonstrated the highest surface values (around 17–18 cm^2^), while surface values generally decreased with increasing pumpkin flour concentration. The 20% enriched sample consistently showed the lowest surface values (14.63 ± 0.8 cm^2^). Statistical analysis revealed highly significant effects (*p* < 0.001) for most factors, including sample, day, and their interactions, while proofing time alone did not show significant effects.

Contrasting findings were found in prior research by Liubych et al., 2023, which revealed that the initial volume of pumpkin-enriched bread is frequently more significant than that of control samples after baking at one hour of proofing [47]. In two-hour proofing, a weaker gluten network was visible, resulting in less gas being trapped, which lowers the volume of the dough after proofing. Różyło et al., 2014 [34] and Davoudi et al., 2020 [48] reported a similar trend with higher concentrations of pumpkin (above 20%), which decreased volume due to the increased density of the dough and potential staling effects. In the study by See et al., 2007 [49], bread containing 5% pumpkin flour demonstrated the highest loaf volume compared to the other samples (control, 10%, 15% pumpkin flour).

Based on the data presented in Table 2, specific volume measurements showed apparent variations across different pumpkin flour concentrations and fermentation times.

In the one-hour fermentation group, the control bread exhibited the highest specific volume (2.25 ± 0.01 cm^3^/g) at day 0, which increased to 2.35 ± 0.03 cm^3^/g after 7 days. A gradual decrease in specific volume was observed with increasing pumpkin flour concentration, with the 20% enriched sample showing the lowest values (1.69 ± 0.03 cm^3^/g at day 0 and 1.80 ± 0.00 cm^3^/g at day 7). In the two-hour fermentation group, the control bread maintained the highest specific volume throughout the storage period, starting at 2.40 ± 0.01 cm^3^/g and slightly decreasing to 2.32 ± 0.00 cm^3^/g after 7 days. All pumpkin-enriched samples showed lower specific volumes than the control, following a similar declining trend with increasing pumpkin flour concentration. The 20% enriched sample again demonstrated the lowest specific volume values (1.69 ± 0.02 cm^3^/g at day 0 and 1.61 ± 0.00 cm^3^/g at day 7).

Statistical analysis indicated highly significant effects (*p* < 0.001) for the day (D), proofing time (P), sample (S), and the interactions A × P and P × S. However, the interactions A × S and A × P × S did not show significant differences (ns), which suggests that both storage time and fermentation duration significantly influence the specific volume of the bread. At the same time, the effect of pumpkin flour concentration remains consistent across different conditions. These observations align with findings in previous studies where adding pumpkin flour decreased specific bread volume; more significant differences were recorded between control and bread containing up to 10% pumpkin flour [29,50]. This decrease in specific volume could be attributed to pumpkin flour’s dilution of the gluten network, which affects the bread’s ability to retain gas during fermentation and baking.

Moreover, the high fiber content in pumpkin flour can interfere with gluten network formation, leading to the collapse of gas-holding cells, which ultimately reduces loaf volume [49]. Liubych et al. (2023) [47] observed an opposite trend, where the specific volume of wheat bread increased by adding 5–20% pumpkin paste. This effect was attributed to the absence of a significant increase in bread mass after enrichment with pumpkin paste within this range. In contrast, See et al. (2007) [49] found that bread containing 5% pumpkin flour had the highest specific volume compared to the control and bread with 10% and 15% pumpkin flour. Similar results were reported by Hoxha et al. (2023) [31], who found that bread enriched with 5% pumpkin flour also achieved the highest specific volume compared to samples with 10%, 15%, and 25% pumpkin flour.

**Figure 1:**
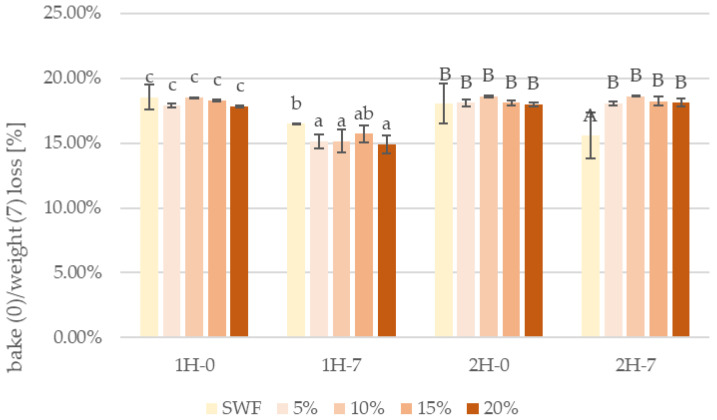
Bake loss of bread right after baking and after 7 days, SWF—soft wheat flour, 5–20%—pumpkin flour addition; 1H, 2H—proofing time; 0, 7—time points of measurement, 0—day of baking, 7—after 7 days respectively. Lowercase letters denote significant differences among 1 h proofed samples at *p* = 0.05; uppercase letters denote significant differences among 2 h proofed samples at *p* = 0.05.

Weight loss of composite breads concerning the proofing time on day 0 and after 7 days of storage is presented in Figure 1.

The bake loss of bread samples was analyzed under different proofing and storage conditions. For one-hour proofed samples (1H), a bake loss of 18% was measured immediately after baking (day 0), significantly decreasing to 15% after 7 days of storage. In two-hour proofed samples (2H), the initial bake loss was measured at 18%, and a high weight loss (17.5%) was maintained after 7 days of storage. This pattern suggests extended proofing time affects the bread’s moisture retention mechanisms during storage. The relationship between proofing time and moisture retention characteristics suggests fundamental differences in crumb structure development between 1H and 2H proofed samples, reflected in their distinct moisture loss patterns during storage. This suggests that extended fermentation time may influence the bread’s moisture retention capabilities during storage [51].

These findings suggest that fermentation time plays a role in the bread’s moisture retention characteristics during storage, with longer fermentation times (2H) resulting in more stable weight loss patterns compared to shorter fermentation times (1H), where a more pronounced decrease in weight loss was observed over the storage period. This could affect the bread’s freshness and texture characteristics during storage.

Water activity and porosity of composite breads concerning the proofing time on day 0 and after 7 days of storage are presented in Table 3. 

For one-hour fermentation (1H), the control bread showed initial water activity of 0.954 ± 0.002, slightly increasing to 0.958 ± 0.006 after 7 days. A gradual decrease in water activity was observed with increasing pumpkin flour concentration, with the 20% enriched sample showing the lowest values (0.945 ± 0.002 at day 0 and 0.941 ± 0.002 at day 7). A similar relationship between water activity and the amount of pumpkin flour in bread was observed in the study by Chikpah et al., 2023 [51] and Wongsagonsup et al. 2015 [52]. This can be attributed to the fibre and sugar content in pumpkin flour, as these components can bind water, reducing the amount of free water available in the food.

In the two-hour fermentation (2H) samples, the control bread exhibited higher initial water activity (0.966 ± 0.002) than the 1H samples, which decreased slightly to 0.962 ± 0.004 after 7 days. Like 1H fermentation, increasing pumpkin flour concentration decreased water activity, with the 20% enriched sample showing the lowest values (0.945 ± 0.004).

For one-hour fermentation, the control bread showed an initial porosity of 55.7 ± 11.6%, which increased significantly to 91.6 ± 3.6% after 7 days. The initial porosity values were similar across different pumpkin flour concentrations at day 0 (ranging from approximately 53–58%). In the study conducted by Păucean and Man (2014) [53], the porosity of wheat bread fermented for 70 min was reduced by adding 15% and 30% pumpkin pulp compared to the control. Similarly, Różyło et al. (2014) [34] observed that bread made with pumpkin pulp exhibited smaller, more compact pores. However, after 7 days, all samples showed increased porosity, ranging from 75.7 ± 6.0% to 91.6 ± 3.6%.

In two-hour fermented samples, the control bread demonstrated notably higher initial porosity (87.8 ± 3.8%) than 1H samples. The porosity generally decreased with increasing pumpkin flour concentration, with the 20% enriched sample showing the lowest initial porosity (49.4 ± 6.1%). After 7 days of storage, most samples showed increased porosity values. 

The relationship between porosity and volume measurements during storage reveals interesting structural changes in the bread samples. For the proofing of 1 h, the control bread exhibited an initial porosity of 55.7 ± 11.6%, dramatically increasing to 91.6 ± 3.6% after 7 days of storage. This substantial increase in porosity correlates with the observed volume changes, where the control bread’s volume increased from 189.9 ± 0.7 cm^3^ to 195.5 ± 1 cm^3^. Similar patterns were observed across different pumpkin flour concentrations and fermentation times. The two-hour fermented samples showed comparable trends, with the control bread demonstrating notably higher initial porosity (87.8 ± 3.8%) than the one-hour fermented samples. 

Cross-sectional analysis of the bread samples reveals that the initially uniform crumb structure undergoes significant changes during storage (Supplementary Figure S1). The crumb simultaneously experiences drying and internal shrinking of the cell walls, leading to internal cracks and separations. 

These structural modifications create internal stresses that slightly expand the overall loaf dimensions, explaining the increased porosity measurements and the apparent volume increase observed during storage. The phenomenon appears more pronounced in samples with higher pumpkin flour content, suggesting that the fibre components may influence the pattern of these structural changes during storage.

The duration of fermentation significantly impacts the bread’s water activity. This implies that prolonged fermentation periods could improve the bread’s capacity to retain moisture, impacting its texture and rate of staling over time. Furthermore, adding pumpkin flour enhances the bread’s nutritional value and helps it retain moisture, as shown by the higher water absorption of loaves added with different amounts of pumpkin flour [54]. According to a prior study, adding 5% to 10% pumpkin flour may significantly enhance the bread’s texture and appearance, producing a more attractive final product with a brilliant yellow colour [40].

Statistical analysis indicated highly significant effects (***) for most parameters and their interactions, suggesting that fermentation time and pumpkin flour concentration significantly influence the bread’s water activity and porosity characteristics.

The texture profile of composite bread concerning the proofing time 1 h and 2 h on day 0 and after 7 days of storage is presented in Table 4.

The hardness of bread samples showed significant variation with storage time and pumpkin flour concentration. For a one-hour proofing time, the control bread exhibited an initial hardness of 6.8 ± 1.5 N, which increased to 32.3 ± 7.1 N after 7 days. Higher pumpkin flour concentrations resulted in greater initial hardness, with the 20% sample reaching 14.3 ± 1.5 N, ultimately increasing to 40.4 ± 3.5 N after 7 days. Similar trends were observed with 2-h proofing, where the initial hardness of the control was higher at 10.3 ± 2.7 N, increasing to 35.8 ± 2.6 N.

The increase in hardness with pumpkin flour addition is consistent across both proofing times. It corroborates previous findings where increased pumpkin content from 0 to 20% led to a linear increase in bread crumb hardness [29,39] attributed to gluten dilution, as gluten plays a critical role in gas cell formation and elasticity [55]. The fibre in pumpkin flour likely interferes with starch-gluten interactions, reducing crumb cohesiveness [51]. Notably, Kampuse et al. (2015) [54] observed that fresh pumpkin pulp initially decreased crumb hardness at lower concentrations but increased it at higher levels (40% and 50%).

Cohesiveness decreased over storage time for all samples, with initial values between 0.71 and 0.83 dropping to 0.49 and 0.56 after 7 days. This decline is linked to the disruption of starch-gluten interactions caused by pumpkin flour’s fibre content [36,42]. Chewiness, a combination of hardness, cohesiveness, and springiness, increased significantly after storage, particularly in the control and 20% pumpkin flour samples. This trend aligns with previous studies where chewiness correlated with the textural changes driven by pumpkin flour addition [34,49].

Springiness remained relatively stable, ranging from 0.66 to 0.82, although a general decrease in springiness with pumpkin flour addition was observed, likely due to reduced gluten content affecting bread elasticity [34,51,56]. Resilience, defined as the bread’s ability to return to its original shape post-compression, decreased across all samples, further indicating the weakening of the gluten network with increased pumpkin flour concentrations [51].

Statistical analysis confirmed highly significant effects (*p* < 0.001) for storage time, sample type, and their interactions in both fermentation conditions, emphasizing that both factors considerably influence bread texture properties. Additionally, the incorporation of pumpkin flour altered bread porosity, resulting in smaller and denser pores, as observed by Różyło et al. (2014) [34], who noted an increase in the Dallmann porosity index. Adding pumpkin flour also reduced bread staling, attributed to its high fiber content, which aids moisture retention [34,51].

Despite these textural changes, the sensory acceptability of pumpkin flour-enriched bread remains high, with consumers generally appreciating the modified texture [26,49,54,57]. Shevchenko et al. (2023) [58] also highlighted that extended fermentation enhances structural integrity and stimulates physicochemical processes, although specific impacts on texture were not detailed.

**Figure 2:**
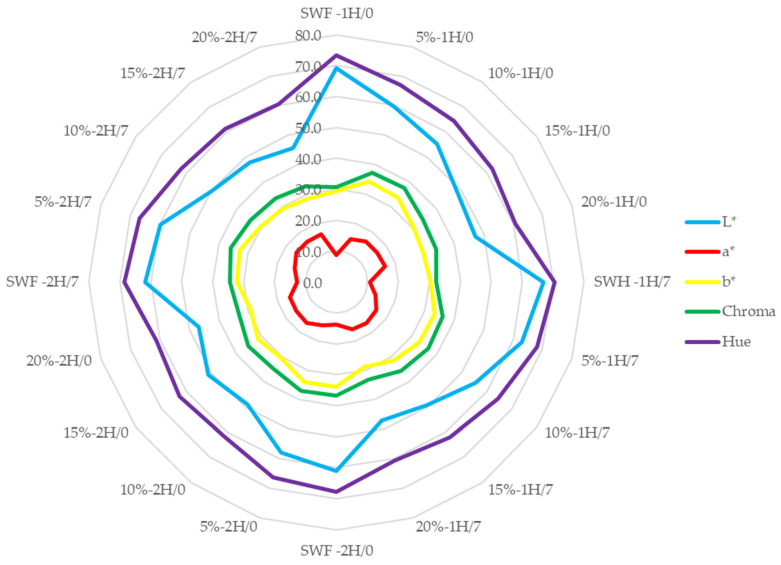
Colour parameters of bread crust. Control—SWF—soft wheat flour, 5%, 10%, 15%, and 20% indicate the percentage of pumpkin flour in the flour. Here, 1 and 2 indicate the proofing time, and 0 and 7 indicate time points in days.

**Figure 3:**
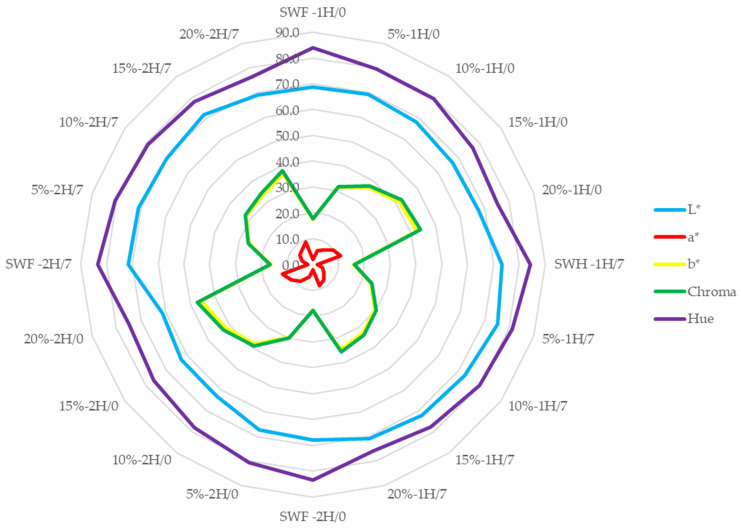
Colour parameters L*, a*, b*, Chroma, and hue angle of bread’s crumb at time 0 and 7 days. 5%, 10%, 15%, and 20% indicate the percentage of pumpkin flour in the breads. Here, 1 and 2 indicate the proofing time.

The color changes profile of composite breads concerning the proofing time on day 0 and after 7 days of storage are presented in Figure 2 (crust) and Figure 3 (crumb).

The colour parameters (L*, a*, b*, chroma, and hue angle) of bread crust under different conditions of fermentation time (1H and 2H), storage period (0 and 7 days), and pumpkin flour concentration (control, 5%, 10%, 15%, and 20%) reflect several distinct patterns.

The lightness (L*) parameter, represented by the blue line, shows relatively high values across all samples, with some variations between different concentrations and fermentation times. The control samples generally exhibited higher L* values, indicating lighter crust colour, while increasing pumpkin flour concentration led to decreased L* values, suggesting darker crust colouration. These findings align with Kampuse et al. (2015) [54] and Chikpah et al. (2023) [51], who noted that replacing wheat flour with pumpkin flour resulted in decreased L* values due to the carotenoids in pumpkin flour. Similarly, See et al. (2007) [49] observed a significant reduction in crust lightness with pumpkin flour addition.

The redness (a*) parameter, shown by the red line, demonstrates relatively low values compared to other parameters, indicating minimal red colouration in the crust. However, a slight increase in a* values with increasing pumpkin flour concentration, particularly in the 15% and 20% samples, is evident. Chikpah et al. (2023) [36] reported that at lower baking temperatures (150–180 °C), a* values increased with pumpkin flour addition, while higher temperatures (≥190 °C) led to decreased a* values. In contrast, See et al. (2007) [49] observed a rise in a* values for crumb with pumpkin flour addition, suggesting that the effect of pumpkin flour on a* depends on baking conditions.

The yellowness (b*) parameter, indicated by the yellow line, shows moderate values that increase with higher pumpkin flour concentrations, reflecting the natural yellow pigmentation contributed by the pumpkin flour. This is consistent with findings from Kampuse et al. (2015) [54], Rakcejeva et al. (2011) [59], and Chikpah et al. (2023) [51], who reported an increase in b* values with pumpkin flour, enhancing the yellow color of both crust and crumb. See et al. (2007) [49] also confirmed similar results, emphasizing that pumpkin flour increases b* values, imparting a stronger yellow color.

The chroma values (green line) follow a pattern similar to the b* values, suggesting that the yellowness component primarily influences the overall color intensity. This is particularly evident in samples with higher pumpkin flour concentrations. Batista et al. (2018) [60] demonstrated that replacing wheat flour with pumpkin seed flour maintained Chroma values despite significant L* reductions, confirming that pumpkin flour intensifies colour saturation.

The angle (purple line) shows the highest values among all parameters, indicating the predominant colour tone of the crust. The variation in hue angle across different samples suggests changes in the overall colour perception with different pumpkin flour concentrations and fermentation times.

These observations align with the findings of previous studies by See et al. (2007) [49], which reported that the crumb colour of the sample significantly increased in redness (a value) and yellowness (b value). This change might be attributed to the yellow pigment imparted by the pumpkin. However, there was a decrease in both the L* value (lightness) and h value (hue) with higher percentages of pumpkin flour addition. The colour change ranged from light brown (control) to darker brown (15%), as loaves containing additional glucose developed a darker crust. Similarly, Ge et al. (2021) [61] noted that lower levels of pumpkin flour (5% and 10%) produced a vibrant yellow color in bread crumbs, positively influencing consumer acceptability.

The color parameters (L*, a*, b*, Chroma, and Hue angle) of bread crumbs across different pumpkin flour concentrations (control, 5%, 10%, 15%, and 20%), fermentation times (1H and 2H), and storage periods (0 and 7 days) showed several distinctive patterns. The lightness (L*) parameter demonstrates higher values than the crust measurements, indicating generally lighter colouration in the crumb. The L* values gradually decrease with increasing pumpkin flour concentration, suggesting that higher pumpkin content results in a darker crumb color. This trend is consistent with findings from Kampuse et al. (2015) [54] and Chikpah et al. (2023) [51], confirming that pumpkin flour addition reduces crumb lightness due to the natural pigments in pumpkin.

The redness (a*) parameter shows notably lower values in the crumb than the crust, indicating minimal red colouration, even with increasing pumpkin flour concentrations. Similar findings were reported by See et al. (2007) [49], who observed a rise in crumb a* values with pumpkin flour, although this effect still needs to be improved in most samples.

The yellowness (b*) and chroma values show similar patterns, both increasing with higher pumpkin flour concentrations, suggesting that pumpkin flour significantly contributes to the yellow colouration of the crumb, affecting overall colour intensity. Rakcejeva et al. (2011) [59] found that b* values in crumbs with dried pumpkin flour were 1.23 times higher than in control samples, corroborating these observations. Chikpah et al. (2023) [51] also confirmed that pumpkin flour enhances b* values in crust and crumb, intensifying yellow.

The hue angle demonstrates the highest values among all parameters, indicating a more consistent pattern than the crust measurements. This implies a more uniform color distribution in the crumb compared to the crust. However, further studies are needed to confirm the specific effects on hue.

**Figure 4:**
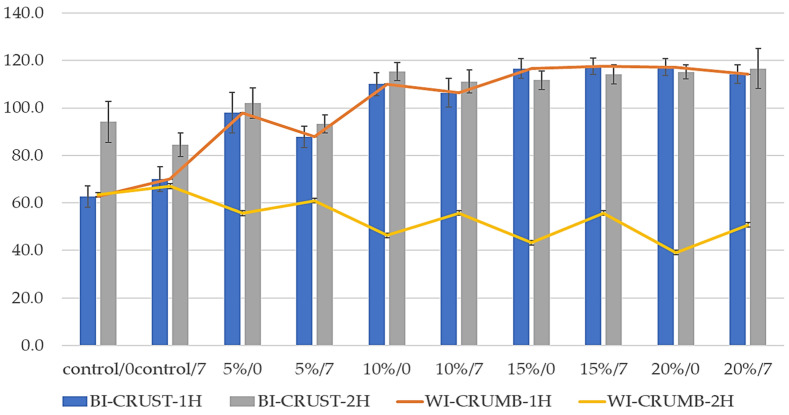
Browning Index and Whitening Index of bread’s crust and crumb at 0 and 7 days; 5%, 10%, 15%, and 20% indicate the percentage of pumpkin flour in the flour. 1 H and 2 H indicate the proofing time; 0, 7—time points of measurement, 0—day of baking, and 7—after 7 days, respectively.

The browning and whitening indexes of composite bread concerning the proofing time on day 0 and after 7 days of storage are presented in Figure 4.

The Browning Index for the crust (BI-CRUST) shows different patterns between 1H and 2H fermentation times. In 1H fermentation (blue bars), there is a general trend of increasing BI values with higher pumpkin flour concentrations, from approximately 60 units in the control to around 115 units in the 20% samples. The 2H fermentation samples (grey bars) show a similar increasing trend but generally higher initial values, particularly in the control samples. The observed increase in browning with pumpkin flour concentration can be attributed to natural sugars and carotenoids in pumpkin, which enhance the Maillard reaction during baking, resulting in a darker crust. This is supported by Erbaş et al. (2012) [62], who found that higher baking temperatures boost the Maillard reaction, leading to more intense browning. Additionally, Chhanwal and Anandharamakrishnan (2014) [63] noted that the browning index rises when the bread surface temperature surpasses 120 °C, aligning with the increased browning observed in higher pumpkin flour concentrations.

Some studies indicate that adding pumpkin flour can significantly impact the browning index of bread. For example, Erbaş et al. (2012) [62] highlighted that the natural sugars in pumpkin interact with proteins during baking, intensifying the browning process. This aligns with Chhanwal and Anandharamakrishnan’s (2014) [63] findings, who observed that the browning index increases substantially with surface temperatures above 120 °C. The enhanced browning effect in the crust can be explained by the combined effect of natural sugars and carotenoids in pumpkin flour, contributing to a darker and more appealing crust coloration.

The Whitening Index for the crumb (WI-CRUMB) displays contrasting patterns between fermentation times. The 1H fermentation samples (orange line) show an increasing trend with higher pumpkin flour concentrations, starting from around 65 units in the control and reaching approximately 120 units in the 15–20% samples. Conversely, the 2H fermentation samples (yellow line) show a slight decreasing trend with increasing pumpkin flour concentration, ranging from about 65 units in the control to 45 units in the 20% samples. This suggests that a shorter fermentation time (1H) allows for better preservation of crumb lightness. In contrast, longer fermentation times (2H) might intensify enzymatic browning processes or enhance pigment release from pumpkin flour, decreasing crumb lightness.

The error bars Indicate some varia”Ilit’ In measurements, particularly in the 2H fermentation samples, suggesting that longer fermentation times may lead to more variable browning and whitening patterns. This variability may be due to differences in enzymatic activity or moisture content during longer proofing periods, influencing crust coloration more unpredictably. Additionally, the contrasting trends between crust and crumb indices highlight that adding pumpkin flour affects the bread’s exterior and interior differently. Maillard reactions and caramelization predominantly drive crust browning, while pigment stability and enzymatic browning may influence crumb whitening or darkening. This dual effect can be advantageous for developing bread with distinct visual appeal and texture, where a darker crust enhances consumer perception of artisan-style bread while maintaining a visually lighter crumb for internal contrast.

**Figure 5:**
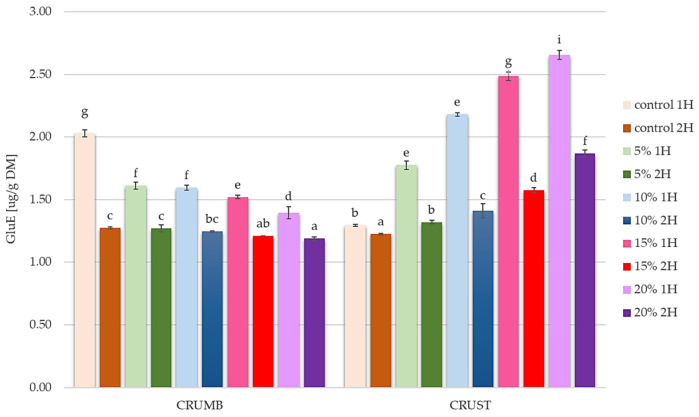
Reducing sugars content in the bread’s crust and crumb at 1H and 2H proofing at day 0; 5%, 10%, 15%, and 20%—pumpkin flour share in composite bread; GlueE—glucose equivalent; DM—dry matter; lowercase letters denote significant differences among samples in crumb and crust groups at *p* = 0.05.

The reducing sugar content in composite bread concerning different proofing time time 1 h and 2 h on day 0 is presented in Figure 5.

As shown in Figure 5, which presents the reduced sugar content in bread crust and crumb under different fermentation times (1H and 2H), a clear relationship can be observed between sugar content, pumpkin flour concentration, proofing time, and the colour parameters previously discussed in Figure 2 and Figure 3.

The reducing sugars content showed a decreasing trend with higher pumpkin flour concentrations in the crumb, while increasing in the crust. This pattern directly correlates with the Browning Index (BI) results from Figure 4, where higher pumpkin flour concentrations led to increased browning, particularly in the crust. This correlation can be explained by the higher availability of reducing sugars in Maillard reactions during baking, contributing to enhanced browning.

When examining the relationship between reducing sugars and colour parameters from Figure 3, the increased reduced sugar content aligns with the observed changes in L* values (decreased lightness) and a* values (increased redness) in the crumb. This suggests that more reducing sugars not only affect the crust browning but also influence the internal colour development of the bread.

Research has shown that incorporating pumpkin flour can enhance the levels of reducing sugars, as complex carbohydrates break down during baking. For example, Yu et al. (2021) found that the degradation of pumpkin polysaccharides during processing produces small-molecule reducing sugars, which can enhance the sweetness and browning of the bread [64]. This is supported by Ma et al. (2020), who observed that adding pumpkin flour to bread recipes increased the reducing sugar content due to the hydrolysis of pumpkin polysaccharides during the baking process [55].

The correlation between reducing sugar content and color development appears more pronounced in 1H fermentation samples compared to 2H fermentation, suggesting that fermentation time influences the relationship between sugar availability and color development. This observation helps explain the variations in both browning and whitening indices observed between different fermentation times in Figure 4.

**Figure 6:**
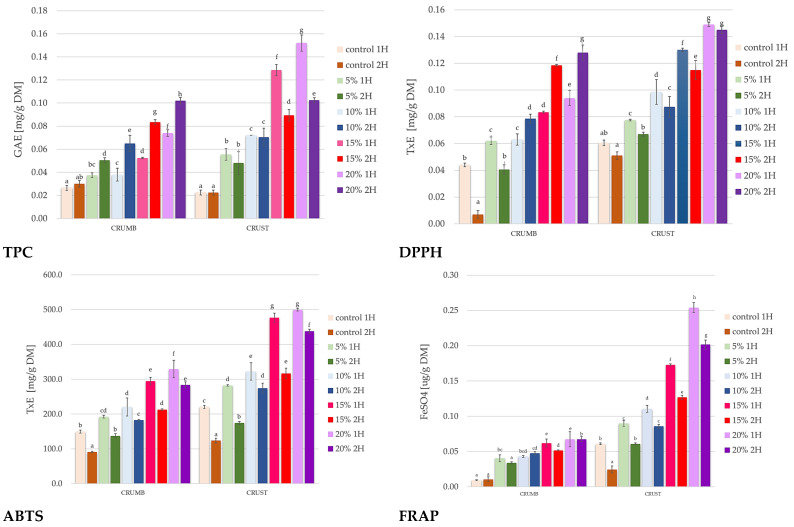
Total polyphenol content (TPC), antioxidant activity vs. DPPH and ABTS, and reducing power (FRAP) in composite bread concerning the proofing time on day, 1H, 2H—proofing time; 5%, 10%, 15%, and 20%—pumpkin flour share in composite bread; TE—Trolox glucose equivalent; GAE—gallic acid glucose equivalent; DM—dry matter; lowercase letters denote significant differences among samples in crumb and crust groups at *p* = 0.05.

The total polyphenol content (TPC), antioxidant activity vs. DPPH and ABTS, and reducing power (FRAP) of the crumb and crust of composite bread concerning the proofing time 1 h and 2 h on day 0 is shown in Figure 6.

Figure 6 shows higher pumpkin flour concentrations in the crust and increased crumb polyphenol content. This trend is more pronounced in the crust, likely due to the concentration of compounds during baking. Research indicates that incorporating pumpkin flour into bread enhances its polyphenol content. Wahyono et al. (2020) discovered that as the amount of pumpkin flour increased, the total phenolic content rose significantly, peaking in bread made with 20% pumpkin flour [65]. Another study by Akbaş and Kılmaoğlu (2022) found that incorporating pumpkin powder into the bread formulation increased the phenolic content when 10% pumpkin was used [66]. Also, in studies conducted on the bioactive properties of *Cucurbita moschata*, one of the varieties of yellow pumpkin from the Cucurbitaceae family, it was confirmed that 10% and 15% addition of yellow pumpkin flour increased the total content of polyphenols in pita bread. The same study also found that a 5% and 15% addition of yellow pumpkin fruit flour increased the anti-radical activity determined by the DPPH method [67].

In our study, extracts obtained from composite bread after 2 h proofing showed a lower polyphenolic compound content than bread immediately after baking. It was confirmed that the most noticeable decreases in the total content of phenolic compounds (present in products such as cereal flakes, edible flowers, fruits, nuts, and seeds) occur as a result of exposure to sunlight at temperatures in the range of 23–40 °C [68]. However, phenolic compounds such as quercetin, gallic acid, sinapic acid, and p-hydroxybenzoic acid, found in pumpkin fruits, can readily undergo oxidative degradation [68]. The results of similar studies showed that adding pumpkin seed flour to the mixture of ingredients necessary for preparing wafers (such as flour, water, oil, milk powder, ammonium carbonate, baking powder, whey, and lecithin) increases the content of polyphenolic compounds in the wafers [69] and was confirmed by Aljobair (2024) in studies in which dried peels of green pumpkin, watermelon, and cucumber were mixed to obtain a powder from the peels of these raw materials, which was used to produce functional bread [70].

Our studies showed that in the case of composite bread with 15% and 20% pumpkin flour content, storage for 2 h proofing resulted in a much more significant reduction in the total phenolic content than in the case of composite bread with 5% pumpkin content. This may have been related to the fact that flavonoids in pumpkin flour are a fraction of polyphenolic compounds more susceptible to degradation during storage due to temperature or oxygen exposure [71]. Therefore, the flavonoids in the bread with a lower pumpkin flour content (5%) could be more protected by the proportionally higher amounts of polysaccharides, such as starch or indigestible dietary fiber from wheat, than the flavonoids in the bread with a higher pumpkin flour content (15% and 20%). Also, in the studies conducted by Aljobair (2024), it was shown that the anti-radical activity against DPPH radicals was higher in bread containing dried peels of green pumpkin, watermelon, and cucumber (as a mixture) compared to the control bread [70].

Our studies have shown that the content of polyphenolic compounds in extracts obtained from composite bread increases compared to the control (bread without pumpkin flour) in proportion to the added pumpkin flour. In addition, the bread crumb from baking dough subjected to two-hour fermentation was characterized by a higher value of the total polyphenolic compounds than the bread crumb resulting from baking dough after one-hour fermentation. It has been confirmed so far that phenolic compounds (present in raw materials such as cereal flakes, edible flowers, fruits, nuts and seeds) are susceptible to degradation under the influence of environmental factors such as sunlight and high temperatures, even in the range of 23–40 °C [69]. 

On the other hand, research confirms that pumpkin flour is rich in sugar substances (including simple sugars) and amino acids (including serine and methionine), which may undergo the Maillard reaction when baking bread at high temperatures [72]. In turn, Maillard reaction products, including low and high molecular weight melanoidin fractions, as well as directly reducing sugar and amino acid molecules, can also react with the Folin–Ciocalteu reagent used to measure total polyphenolic content [73]. This could have caused an overestimation of the total polyphenolic content, which occurred in the bread crumb resulting from baking the dough after two-hour fermentation. This is also confirmed by the reduced content of reducing sugars observed in bread made from dough after two hours of fermentation and characterized by adding pumpkin flour in the range of 5–20% (Figure 5).

In our study, it was observed that the antioxidant activity in the crust of composite bread increased proportionally with the increase in pumpkin flour addition (mainly in the case of the DPPH method in the range of 5–20%) and increased proportionally in the crumb, in the range of 10–20% pumpkin flour addition in the case of the ABTS method. Moreover, in the case of the ABTS method, in the full range of 5–20% pumpkin flour addition, the antioxidant activity was higher both in the crust and in the crumb of bread made from dough after 1 hour of fermentation than in bread made from dough after 2 hours of fermentation.

This may be because antioxidants such as β-carotene and α-tocopherol are intensively released from pumpkin flour only in the initial period of dough fermentation, while the release process of these substances is inhibited in the second hour of fermentation. Moreover, the mentioned antioxidant compounds such as β-carotene could have been oxidized in the second hour of fermentation, which would explain the occurrence of lower antioxidant activity of individual bread samples with the addition of pumpkin flour compared to the composite bread made from dough after one hour of fermentation [74,75].

The increase in the reduced capacity of the obtained composite bread from soft wheat due to the addition of pumpkin fruit flour may also be related to many minerals in pumpkin fruits, such as potassium, magnesium, iron and selenium [65]. In our studies, the reducing activity increased proportionally (compared to the control bread) with the increase in pumpkin fruit flour. The higher reducing activity we obtained in the crust than in the bread crumb could be caused by the higher concentration of reducing substances, such as phenolic acids, in the outer part of the composite bread. Some phenolic acids, such as syringic acid, cinnamic acid, and protocatechuic acid, and polyphenols, such as p-cumaroylhexoside, quercetin glucoside, and vanillin, are known as potent antioxidants and reducing substances [76]. These substances are probably released from the bread matrix during extraction to a greater extent from the crust than from the bread crumb, where the concentration of compounds with reducing properties is lower.

The correlations among these measurements suggest a strong relationship between polyphenol content and antioxidant activity. Higher polyphenol content consistently corresponds with increased antioxidant capacity across all three measurement methods (DPPH, ABTS, and FRAP). This correlation is particularly evident in samples with higher pumpkin flour concentrations (15–20%).

These findings align with previous research studies. Davoudi et al. (2020) found that pumpkin powder enhanced bread’s sensory qualities and improved its antioxidant properties, indicating a positive relationship between the amount of pumpkin used and its antioxidant activity [48]. Kampuse et al. (2015) showed that incorporating pumpkin by-products into wheat bread significantly increased total carotenoids, recognized for their antioxidant benefits [54]. This is especially important since carotenoids can effectively neutralize free radicals, thus improving the overall antioxidant profile of the bread.

The correlation between fermentation time and antioxidant properties suggests that longer fermentation (2H) generally results in slightly lower antioxidant activity than shorter fermentation (1H), possibly due to the degradation of some bioactive compounds during extended fermentation. This pattern is consistent across all three antioxidant measurement methods and corresponds with the trends observed in polyphenol content. The more potent antioxidant activity observed in the crust compared to the crumb might be attributed to the concentration of compounds during baking and the formation of Maillard reaction products known to possess antioxidant properties. This relationship is particularly evident when comparing the FRAP values between crust and crumb samples. 

External appearance and cross sections of composite breads are shown in Supplementary Figure S1. 

**Figure 7:**
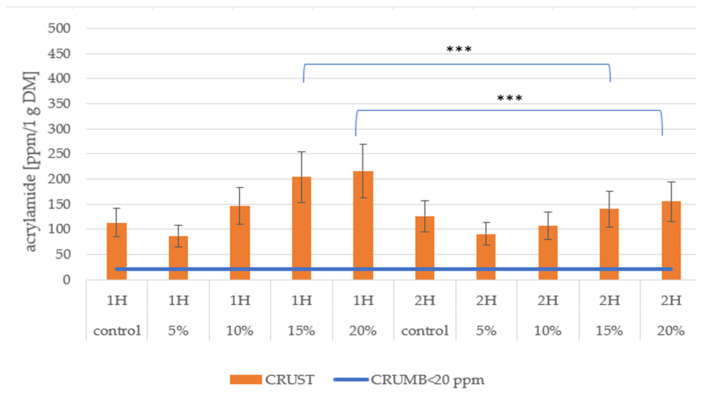
Acrylamide content in the bread’s crust and crumb at 1H and 2H proofing; 5%, 10%, 15%, and 20% indicate the percentage of pumpkin flour in bread. ***—statistically different for *p* < 0.001.

The acrylamide content in the crumb and crust of composite bread concerning the proofing time is presented in Figure 7.

In samples with one-hour fermentation, the acrylamide content in the crust demonstrates a pronounced increasing trend with higher pumpkin flour concentrations. The control samples show approximately 110 ppm, followed by a gradual increase through 5% (90 ppm) and 10% (150 ppm) pumpkin flour addition. A significant spike is observed in 15% and 20% samples, reaching 200–220 ppm, with these differences being statistically significant (***). In contrast, two-hour fermentation samples generally exhibit lower acrylamide levels, with control samples around 130 ppm and a more moderate increase with pumpkin flour addition. The 15% and 20% samples in 2H fermentation show notably lower values (140–150 ppm) than their 1H counterparts, with statistical significance (***) indicating reliable differences. The crumb consistently shows very low acrylamide levels (<20 ppm) across all samples, regardless of fermentation time or pumpkin flour concentration.

When correlating these results with colour parameters from Figure 3, higher acrylamide content in 1H fermentation corresponds with increased a* values (redness) and decreased L* values (lightness), suggesting that more intense colour development aligns with higher acrylamide formation. The Browning Index (Figure 4) showed that increased BI in samples with higher pumpkin flour content directly correlates with higher acrylamide formation, with this relationship being more pronounced in 1H fermentation samples. Additionally, Figure 5 revealed that higher reducing sugar content in samples with more pumpkin flour corresponds to increased acrylamide formation, particularly in the crust, where both parameters show similar increasing trends.

The reduction in acrylamide formation during extended proofing can be attributed to several interconnected mechanisms. The primary mechanism involves the consumption of free asparagine, a crucial precursor for acrylamide formation, by yeast cells during the prolonged fermentation process. During the two-hour proofing period, *Saccharomyces cerevisiae* actively metabolizes asparagine as a nitrogen source, effectively reducing its availability for the Maillard reaction during baking. This is evidenced by the significantly lower acrylamide levels (150 ppm versus 220 ppm) observed in bread with 20% pumpkin flour after 120 min of proofing compared to 60 min.

The extended fermentation time also allows for the enhanced enzymatic activity of asparaginase in the yeast, which hydrolyzes asparagine into aspartic acid and ammonia, further depleting the acrylamide precursor pool. The reduced sugar analysis results also suggest that prolonged proofing leads to increased sugar consumption by yeast, potentially limiting another key reactant in acrylamide formation. The higher moisture retention observed in two-hour proofed samples (water activity 0.966 ± 0.002 versus 0.954 ± 0.002 in control) may also contribute to reduced acrylamide formation by affecting the kinetics of the Maillard reaction during baking.

**Figure 8:**
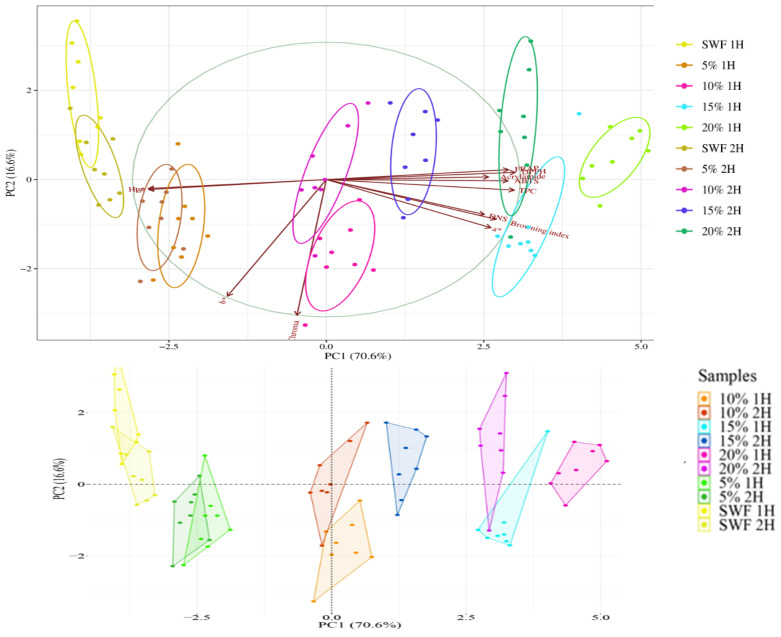
Principal component analysis biplot (**up**) and factor map (**down**) denoted by PC1 and PC2 with a total data variance of 87.2%. PC1 and PC2 are the first and the second principal components (explain the extent of latent variable to the differences). Points represent samples, and different colours represent different groups. Ellipses represent 68% confidence intervals of core regions. Arrows represent original variables, the directions of arrows represent the correlation between the original variable and principal components, and lengths represent the devotion of original data to principal components. A factor map of the PCA was performed on 80 samples and 12 variables. Ten cluster groups corresponding to specific samples were identified.

The PCA plot in Figure 8 shows a data variance of 87.2%. The variance is represented majorly by two factors – PC1 (70.6%) and PC2 (16.6%). Most of the samples were represented by the PC1 factor described by the following equation: 0.291665 × a* + 0.322008 × ABTS + 0.28763 × Acrylamide – 0.175328 × b* + 0.300789 × Browning index – 0.0520891 × Chroma + 0.280108 × DNS + 0.335046 × DPPH + 0.323021 × FRAP – 0.31507 × Hue – 0.315064 × L* + 0.333153 × TPC.

PC1 characterized all samples except the 10% 1H sample. The majority of active variables, such as acrylamide content, reducing sugars content, TPC, ABTS, FRAP, browning index, a*, DPPH, and hue, were characterized by the PC1 dimensional factor. The variables that are adjacent to each other had a close relationship, such as bioactive indicators (TPC, DPPH, ABTS, FRAP, and acrylamide) or dark colour contributors (browning index, a* and reducing sugars), while the observations opposite to each other had a negative correlation with the rest, such as hue, b*, and chroma.

In our studies, both in the crumb and, to an even greater extent, in the crust of composite bread with the highest 20% addition of pumpkin flour, a high total content of polyphenolic compounds was observed, which was correlated with a high content of acrylamide (primarily in the crust). Similar results were obtained in composite bread by Gawlik-Dziki (2009), indicating that the formation of a large amount of Maillard reaction products in bread can increase the in vitro value of the phenolic compounds content in the tested product, which is the reason for the increase in the overall bioactivity in composite and traditional bread [77].

Research has repeatedly found that the highest acrylamide content in bread is in the crust, with research suggesting that approximately 99% of acrylamide is in this layer. Still, the crumb mostly carries negligible quantities [78]. A further contributor to acrylamide levels is the regulator of the fermentation time [79]. Reference has been drawn that prolonging the fermentation period from 1 h (1H) to 2 h (2H) can reduce acrylamide. The research of Rwubatse (2023) [79] showed that an interplay of both long times of fermentation and specific additives resulted in less acrylamide in bread. This effect can be due to the consumption of precursors such as asparagine by the yeasts and bacteria during fermentation; thus, barely acrylamide is formed during baking [80].

However, another study by Jaworska et al. (2019) [81] demonstrated that while some vegetable additions reduced acrylamide formation, pumpkin resulted in higher levels that exceeded safety limits despite increased antioxidant activity. This suggests a complex relationship between pumpkin flour addition, fermentation time, and acrylamide formation that requires careful consideration in bread formulation.

## 4. Conclusions

This study examines the effects of pumpkin flour concentration and proofing time on bread’s physicochemical properties, functional characteristics, and acrylamide content. The findings demonstrate that two-hour proofing combined with 5–10% pumpkin flour substitution achieves an optimal balance between nutritional value and physical characteristics while effectively reducing acrylamide formation. Lower pumpkin flour concentrations maintain acceptable bread quality parameters, while concentrations above 15% negatively impact specific volume and increase crumb firmness. Two-hour proofing shows superior volume retention compared to one-hour proofing, particularly in samples with higher pumpkin flour content.

Water activity decreases with increased pumpkin flour content, while two-hour proofing enhances moisture retention, improving bread stability during storage. Adding pumpkin flour results in darker crust coloration and enhanced yellow hues in the crumb, correlating with higher browning index values and increased reducing sugar content. These changes significantly influence both sensory characteristics and acrylamide formation.

Extended proofing time effectively reduces acrylamide formation while maintaining nutritional benefits. Pumpkin flour enrichment enhances functional properties through increased total polyphenol content and antioxidant activity, as confirmed by DPPH, ABTS, and FRAP measurements. Standardization of two-hour proofing and 5–10% pumpkin flour substitution is recommended for industrial implementation, carefully monitoring color development and reducing sugar content as quality control parameters.

Future research directions should focus on shelf-life extension methods, automation of large-scale production processes, and consumer acceptance studies. Additionally, investigation of other vegetable flour combinations for enhanced anti-acrylamide effects and the development of appropriate packaging solutions would provide valuable insights for industrial applications.

## Figures and Tables

**Table 1:** Volume and surface of the bread after 1 h and 2 h proofing, right after baking, and after 7 days.

	Volume [cm^3^]				Surface [cm^2^]			
Proofing	1H		2H		1H		2H	
Sample	0	7	0	7	0	7	0	7
SWF	189.9 ± 0.7 ^d^	195.5 ± 1 ^d^	178.5 ± 1.4 ^d^	195.1 ± 1.4 ^e^	18.16 ± 0.1 ^d^	17.81 ± 42.8 ^a^	17.16 ± 0.11 ^d^	18.5 ± 0.05 ^d^
5%	168.8 ± 2.7 ^c^	171.9 ± 2.2 ^c^	172.1 ± 0.4 ^c^	181.8 ± 1.6 ^d^	16.91 ± 0.09 ^c^	16.78 ± 26.8 ^b^	16.9 ± 0.04 ^a^	17.58 ± 0.13 ^c^
10%	182.1 ± 1.4 ^d^	176.9 ± 3.8 ^c^	169.9 ± 1.4 ^c^	172.7 ± 3.3 ^c^	17.73 ± 0.06 ^d^	17.2 ± 13.2 ^c^	16.51 ± 0.28 ^cd^	17.01 ± 0.25 ^b^
15%	156.5 ± 9.2 ^b^	154.9 ± 2 ^b^	151.5 ± 0.1 ^b^	162.3 ± 2.5 ^b^	16.02 ± 0.59 ^b^	15.86 ± 3.1 ^d^	15.62 ± 0.06 ^c^	16.46 ± 0.11 ^a^
20%	135.5 ± 0.1 ^a^	140.5 ± 1.5 ^a^	137.3 ± 3.2 ^a^	145.4 ± 0.8 ^a^	14.63 ± 0.8 ^a^	14.82 ± 0.04 ^a^	14.51 ± 0.48 ^b^	16.31 ± 0.11 ^a^
Sample (S)	***		***		***		***	
Day (D)	***		***		***		***	
Proofing (P)	ns		ns		ns		ns	
S × P	ns		ns		ns		ns	
D × P	***		***		***		***	
S × D	***		***		***		***	
S × P × D	***		***		***		***	

SWF—soft wheat flour, 5–20%—pumpkin flour addition; 1H, 2H—proofing time of 1 h and 2 h, respectively; 0, 7—time points of measurement, 0—day of baking, 7—after 7 days respectively; lower-case letters mean values in particular column rows are statistically different (*p* = 0.05). ***—statistically different at *p* < 0.001, ns—statistically non-different at *p* = 0.05.

**Table 2:** The specific volume of the bread in 1H proofing and 2H proofing, right after baking and after 7 days.

	Specific Volume [cm^3^/g]			
Proofing	1H		2H	
Sample	0	7	0	7
SWF	2.25 ± 0.01 ^d^	2.35 ± 0.03 ^e^	2.40 ± 0.01 ^d^	2.32 ± 0.00 ^e^
5%	2.13 ± 0.01 ^c^	2.25 ± 0.02 ^d^	2.07 ± 0.03 ^c^	2.03 ± 0.01 ^c^
10%	2.12 ± 0.03 ^c^	2.15 ± 0.04 ^c^	2.12 ± 0.06 ^c^	2.17 ± 0.01 ^d^
15%	1.87 ± 0.01 ^b^	2.01 ± 0.01 ^b^	1.88 ± 0.02 ^b^	1.89 ± 0.12 ^b^
20%	1.69 ± 0.03 ^a^	1.80 ± 0.00 ^a^	1.69 ± 0.02 ^a^	1.61 ± 0.00 ^a^
Sample (S)	***		***	
Day (D)	***		***	
Proofing (P)	***		***	
S × P	***		***	
D × P	ns		ns	
S × D	***		***	
S × P × D	ns		ns	

SWF—soft wheat flour, 5–20%—pumpkin flour addition; 1H, 2H—proofing time of 1 h and 2 h, respectively; 0, 7—time points of measurement, 0—day of baking, 7—after 7 days respectively; lower-case letters mean values in particular column rows are statistically different (*p* = 0.05). ***—statistically different for *p* < 0.001, ns—statistically not different at *p* = 0.5.

**Table 3:** Water activity and porosity of the bread in 1H proofing and 2H proofing, right after baking and after 7 days.

		Water Activity (%)		Porosity (%)	
Sample	Day	Proofing 1H	Proofing 2H	Proofing 1H	Proofing 2H
control	0	0.954 ± 0.002 ^b^	0.966 ± 0.002 ^c^	55.7 ± 11.6 ^a^	87.8 ± 3.8 ^d^
	7	0.958 ± 0.006 ^c^	0.962 ± 0.004 ^c^	91.6 ± 3.6 ^c^	90.4 ± 4.8 ^c^
5%	0	0.958 ± 0.003 ^b^	0.962 ± 0.003 ^c^	53.7 ± 7.8 ^a^	63.3 ± 6.2 ^c^
	7	0.953 ± 0.002 ^b^	0.955 ± 0.003 ^b^	84.1 ± 6.1 ^b^	80.9 ± 4.3 ^a^
10%	0	0.956 ± 0.002 ^b^	0.957 ± 0.003 ^b^	55.8 ± 6.7 ^a^	62.9 ± 5.4 ^c^
	7	0.954 ± 0.001 ^bc^	0.956 ± 0.002 ^b^	74.3 ± 9.6 ^a^	80.8 ± 9.0 ^ab^
15%	0	0.943 ± 0.003 ^a^	0.953 ± 0.003 ^b^	54.6 ± 5.1 ^a^	59.0 ± 6.1 ^b^
	7	0.945 ± 0.002 ^a^	0.953 ± 0.003 ^b^	86.0 ± 5.3 ^b^	78.9 ± 9.2 ^ab^
20%	0	0.945 ± 0.002 ^a^	0.945 ± 0.004 ^a^	58.5 ± 6.1 ^a^	49.4 ± 6.1 ^a^
	7	0.941 ± 0.002 ^a^	0.945 ± 0.004 ^a^	75.7 ± 6.0 ^a^	84.0 ± 5.4 ^b^
sample (S)		***		***	
day (D)		***		***	
proofing (P)		ns		x	
S × P		ns		***	
D × P		***		x	
S × D		***		x	
S × P × D		***		x	

SWF—soft wheat flour, 5–20%—pumpkin flour addition; 1H, 2H—proofing time; 0,7—time points of measurement, 0—day of baking, 7—after 7 days respectively; lower-case letters mean values in particular column rows are statistically different (*p* = 0.05). ***—statistically different for *p* < 0.001, ns—statistically not different at *p* = 0.05, x—no data.

**Table 4:** Texture profile of breads in 1H and 2H proofing, right after baking and after 7 days.

Proofing 1H						
Sample	Day	Hardness [N]	Cohesiveness	Springiness	Chewiness [N]	Resilience
control	0	6.8 ± 1.5 ^a^	0.83 ± 0.06 ^c^	0.82 ± 0.10 ^b^	4.66 ± 1.25 ^ab^	0.45 ± 0.03 ^ab^
	7	32.3 ± 7.1 ^bc^	0.56 ± 0.10 ^a^	0.74 ± 0.03 ^b^	13.39 ± 3.28 ^b^	0.33 ± 0.06 ^a^
5%	0	9.1 ± 0.6 ^b^	0.77 ± 0.02 ^abc^	0.76 ± 0.04 ^ab^	5.40 ± 0.29 ^b^	0.47 ± 0.03 ^b^
	7	31.8 ± 6.9 ^abc^	0.50 ± 0.05 ^a^	0.71 ± 0.01 ^ab^	11.32 ± 2.88 ^ab^	0.30 ± 0.04 ^a^
10%	0	6.6 ± 1.5 ^a^	0.78 ± 0.08 ^bc^	0.72 ± 0.03 ^a^	3.63 ± 0.72 ^a^	0.44 ± 0.04 ^ab^
	7	24.1 ± 4.1 ^a^	0.50 ± 0.05 ^a^	0.66 ± 0.05 ^ab^	8.46 ± 2.03 ^a^	0.31 ± 0.02 ^a^
15%	0	9.8 ± 2.0 ^b^	0.74 ± 0.01 ^ab^	0.69 ± 0.04 ^a^	4.98 ± 1.25 ^ab^	0.42 ± 0.01 ^a^
	7	31.1 ± 6.5 ^ab^	0.51 ± 0.05 ^a^	0.66 ± 0.07 ^a^	10.24 ± 1.95 ^ab^	0.32 ± 0.04 ^a^
20%	0	14.3 ± 1.5 ^c^	0.71 ± 0.02 ^a^	0.71 ± 0.06 ^a^	7.27 ± 1.42 ^c^	0.43 ± 0.03 ^ab^
	7	40.4 ± 3.5 ^c^	0.49 ± 0.04 ^a^	0.72 ± 0.02 ^a^	12.8. ± 1.05 ^b^	0.33 ± 0.03 ^a^
day (D)		***	***	*	***	***
proofing (P)		***	ns	ns	***	ns
sample (S)		***	*	***	***	ns
D × P		ns	ns	ns	*	ns
D × S P × S		ns	ns	ns	ns	ns
D × P × S		ns	ns	ns	ns	ns
Day (D)		ns	ns	ns	ns	ns
**Proofing 2H**						
**Sample**	**Time**	**Hardness [N]**	**Cohesiveness**	**Springiness**	**Chewiness [N]**	**Resilience**
control	0	10.3 ± 2.7 ^ab^	0.78 ± 0.03 ^a^	0.78 ± 0.07 ^a^	6.26 ± 1.95 ^a^	0.47 ± 0.03 ^b^
	7	35.8 ± 2.6 ^a^	0.56 ± 0.03 ^a^	0.76 ± 0.07 ^b^	15.10 ± 1.59 ^b^	0.33 ± 0.02 ^a^
5%	0	10.2 ± 0.8 ^ab^	0.74 ± 0.01 ^a^	0.78 ± 0.05 ^a^	5.91 ± 0.66 ^a^	0.45 ± 0.02 ^ab^
	7	34.3 ± 1.8 ^a^	0.53 ± 0.01 ^a^	0.75 ± 0.04 ^ab^	13.53 ± 0.83 ^ab^	0.32 ± 0.02 ^a^
10%	0	9.9 ± 1.5 ^a^	0.72 ± 0.02 ^a^	0.76 ± 0.04 ^a^	5.37 ± 0.75 ^a^	0.40 ± 0.04 ^a^
	7	35.5 ± 8.1 ^a^	0.55 ± 0.17 ^a^	0.70 ± 0.05 ^ab^	13.05 ± 1.55 ^ab^	0.33 ± 0.10 ^a^
15%	0	11.6 ± 1.9 ^ab^	0.72 ± 0.04 ^a^	0.69 ± 0.08 ^a^	5.70 ± 1.17 ^a^	0.48 ± 0.05 ^b^
	7	38.0 ± 2.3 ^a^	0.44 ± 0.06 ^a^	0.72 ± 0.05 ^ab^	11.85 ± 1.02 ^a^	0.29 ± 0.04 ^a^
20%	0	12.9 ± 2.0 ^b^	0.73 ± 0.11 ^a^	0.71 ± 0.07 ^a^	6.64 ± 1.23 ^a^	0.44 ± 0.06 ^ab^
	7	41.9 ± 8.1 ^a^	0.53 ± 0.21 ^a^	0.68 ± 0.05 ^a^	14.32 ± 2.37 ^b^	0.37 ± 0.15 ^a^
Day (D)		***	***	*	***	***
Proofing (P)		***	ns	ns	***	ns
Sample (S)		***	*	***	***	ns
D × P		ns	ns	ns	*	ns
D × S		ns	ns	ns	ns	ns
P × S		ns	ns	ns	ns	ns
D × P × S		ns	ns	ns	ns	ns

SWF—soft wheat flour, 5–20%—pumpkin flour addition; 1H, 2H—proofing time; 0, 7—time points of measurement, 0—day of baking, 7—after 7 days respectively; lower-case letters mean values in columns are statistically different (*p* = 0.05). ***—statistically different for *p* < 0.001, *—statistically different for *p* < 0.05, ns—statistically not different at *p* < 0.5.

## Data Availability

The original contributions presented in the study are included in the article/Supplementary Material; further inquiries can be directed to the corresponding author.

## Supplementary Materials

The following supporting information can be downloaded at: https://www.mdpi.com/article/10.3390/foods14030437/s1, Figure S1: Bread external images and cross-section scans.
