# Supplementary material for: Prolonged Proofing Modulates the Acrylamide Content, Nutritional and Functional Characteristics of Pumpkin (Cucurbita maxima Plomo) and Soft Wheat Composite Bread
Source: Foods. 2025 Jan 29;14(3):437. doi: 10.3390/foods14030437 (PMC11817606; doi:10.3390/foods14030437)
Supplement: Supplementary file 1 [file foods-14-00437-s001.zip › foods-3394351-supplementary.pdf]

Figure S.1. External and cross-section appearance of pumpkin enriched bread.

Control	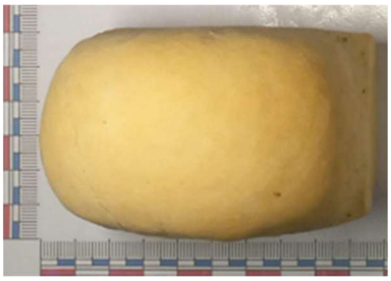	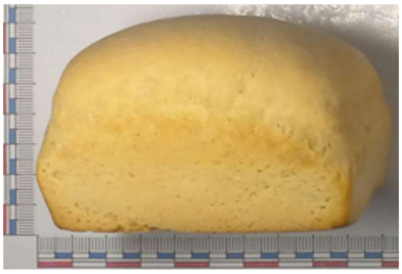	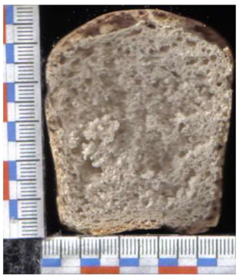
5%	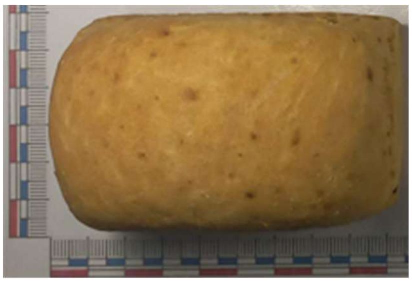	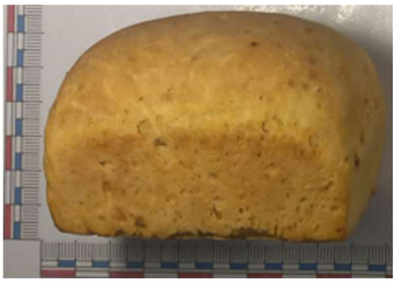	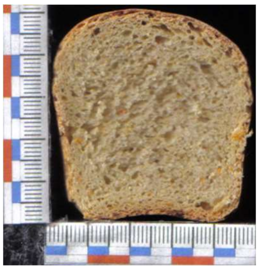
10%	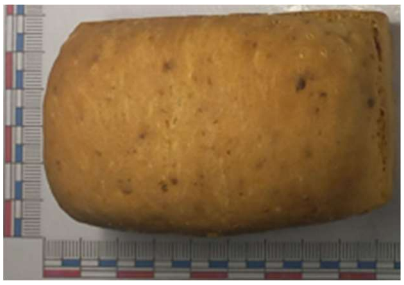	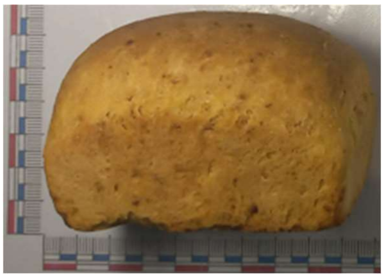	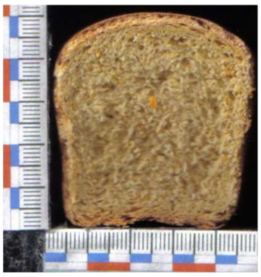
15%	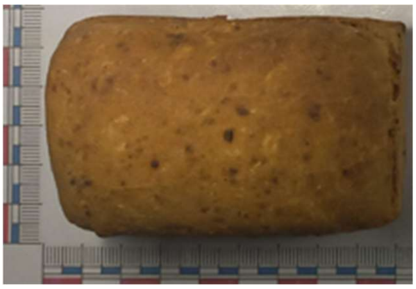	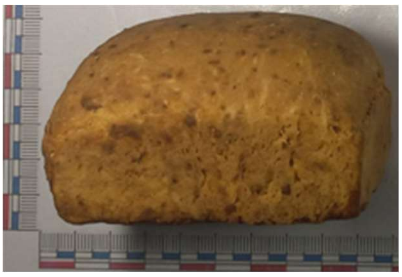	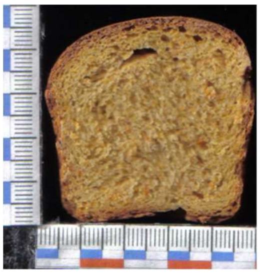
20%	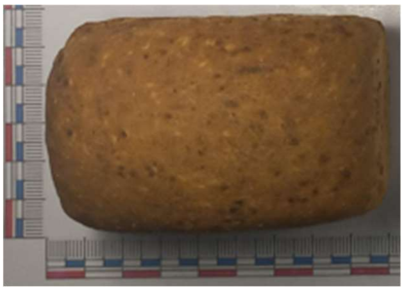	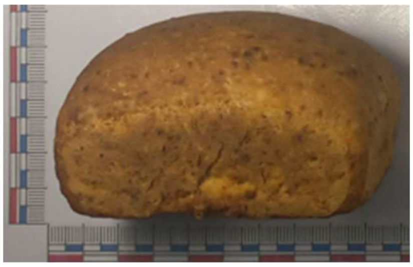	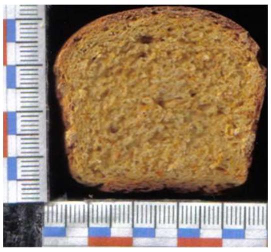

Figure S1. Photos of bread – 0 days – 1H Fermentation.

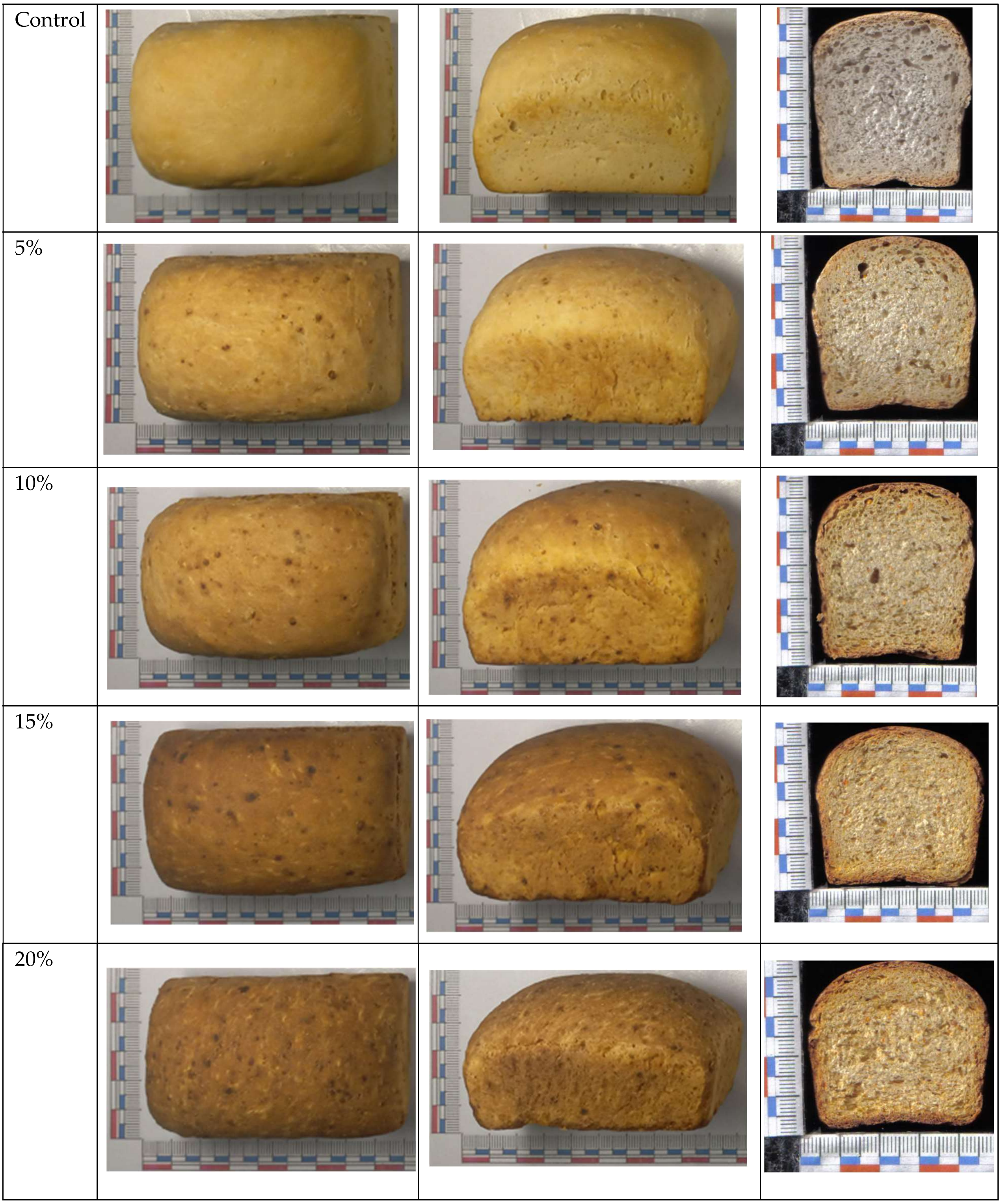

Figure S2. Photos of bread – 7 days – 1H Fermentation.

Control	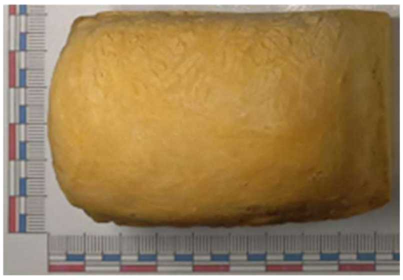	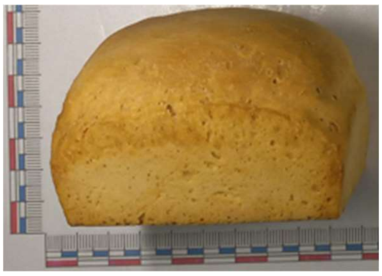	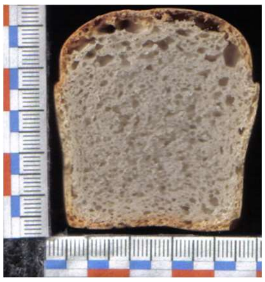
5%	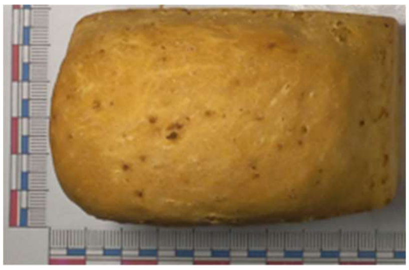	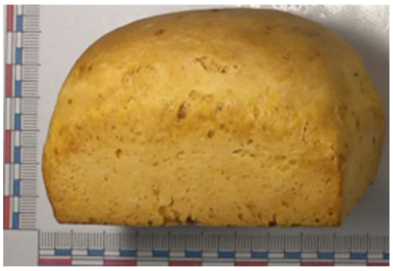	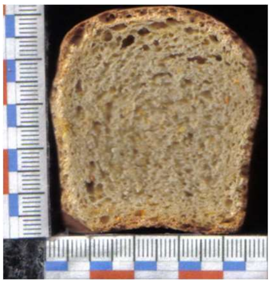
10%	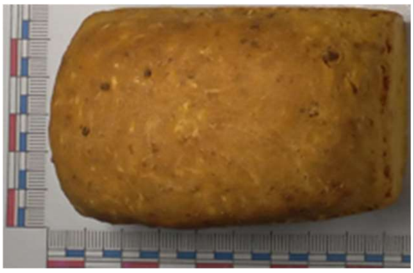	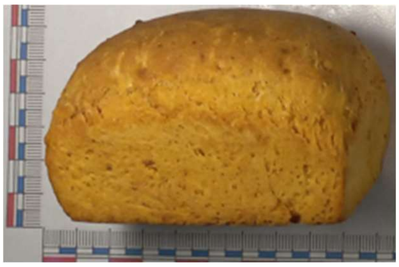	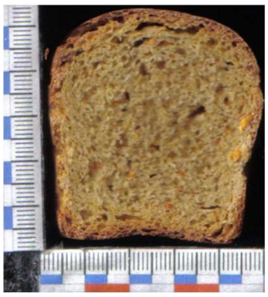
15%	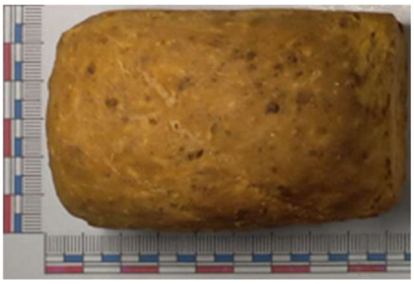	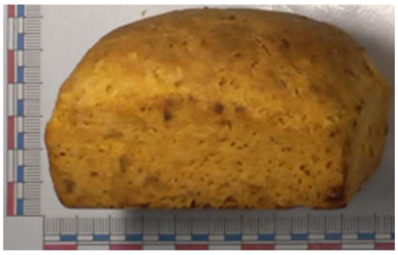	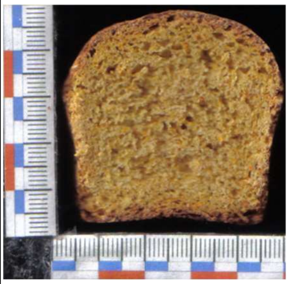
20%	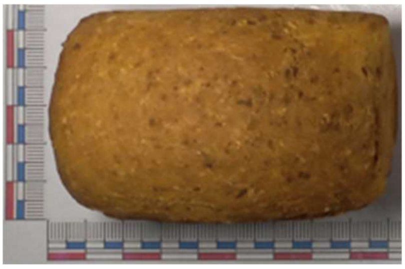	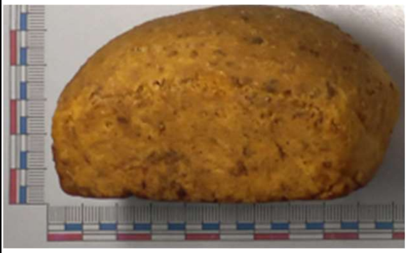	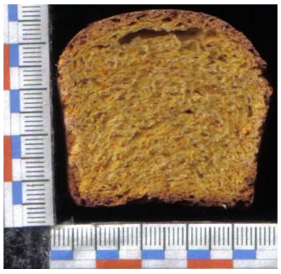

Figure S3. Photos of bread – 0 days – 2H Fermentation.

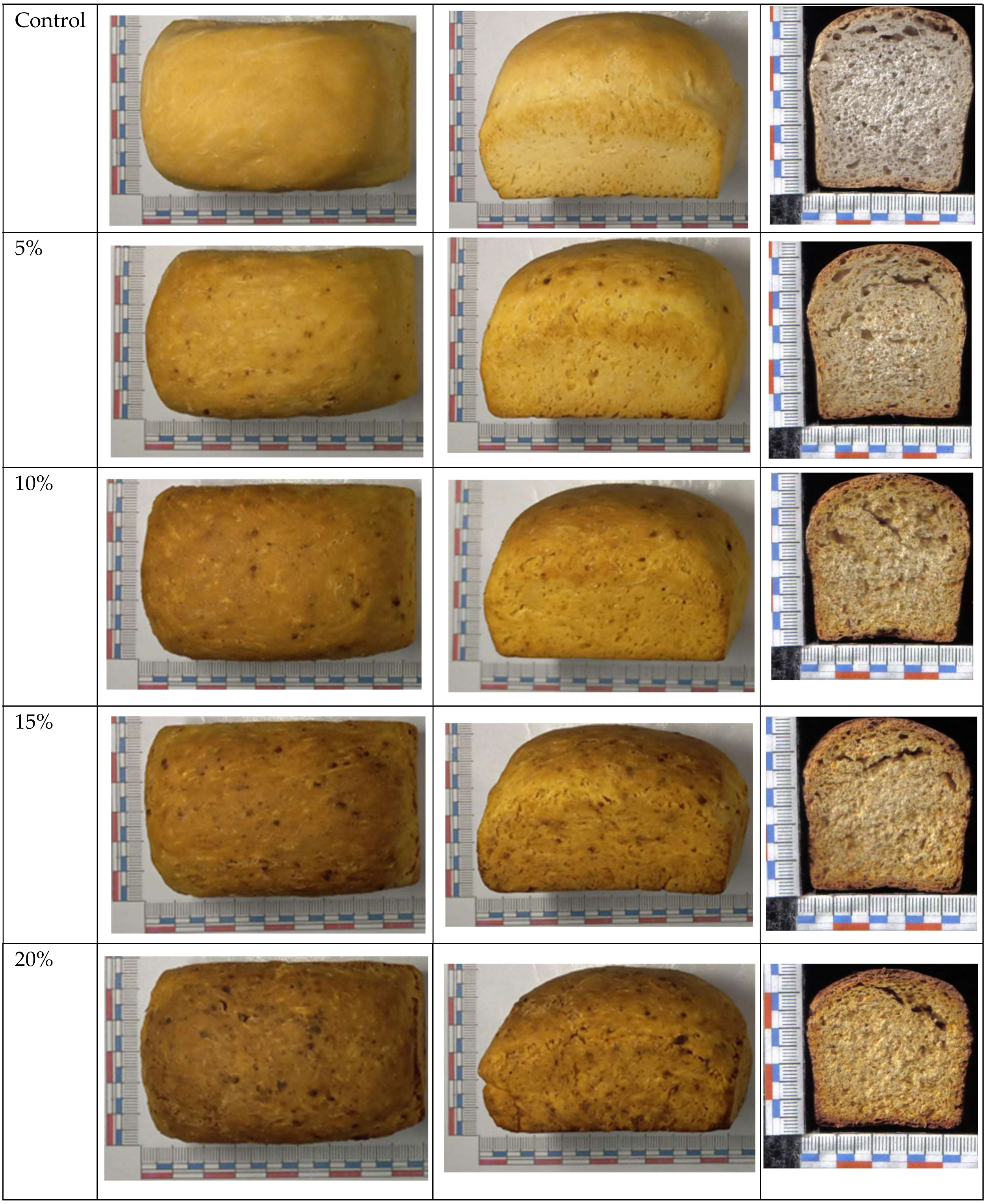

Figure S4. Photos of bread – 7 d
